# Evaluation of LPRDA Pentapeptide for the Prevention and Treatment of *Staphylococcus aureus* Peritoneal Infection

**DOI:** 10.3390/ijms252211926

**Published:** 2024-11-06

**Authors:** Svetlana A. Bozhkova, Ekaterina M. Gordina, Dmitry V. Labutin, Georgy I. Netyl’ko, Polina M. Ivantcova, Konstantin V. Kudryavtsev

**Affiliations:** 1 Vreden National Medical Research Center of Traumatology and Orthopedics, 195427 St. Petersburg, Russia; clinpharm-rniito@yandex.ru (S.A.B.); emgordina@win.rniito.ru (E.M.G.); mailbox@dlabutin.com (D.V.L.); doctornetylko@mail.ru (G.I.N.); 2 Nanobiomedicine Department, Genetics and Life Sciences Centre, Sirius University of Science and Technology, 354340 Sochi, Russia; polina_ivanz@mail.ru

**Keywords:** bacterial resistance, virulence, *S. aureus*, sortase, α-hemolysin, inhibitor

## Abstract

Targeting virulence determinants is a promising approach to controlling *S. aureus* infections in the face of the global spread of antibiotic resistance. *S. aureus*-induced peritonitis often occurs in dialysis, implant and trauma patients. To develop novel prevention and treatment options for peritoneal infection, we investigated the oligopeptide sortase A inhibitor LPRDA as a non-conventional antibacterial that does not affect staphylococcal survival. Administration of LPRDA prior to *S. aureus* challenge reduced the bacterial load of internal organs and bacterial colonization of the abdominal cavity in animals. In addition, LPRDA inhibited α-hemolysin production in 80% of the 35 reference and clinical *S. aureus* strains tested. Consequent research of LPRDA interactions with cefazolin and vancomycin has demonstrated the potential for combined application of the antivirulent and antibiotic agents under study.

## 1. Introduction

Peritonitis typically results from touch contamination with gram-positive organisms under peritoneal dialysis (PD) conditions. As a cause of death in more than 10% of PD patients and an important factor in the termination of PD, peritonitis poses a risk for the development of encapsulating peritoneal sclerosis (EPS), decreased residual renal function, peritoneal fibrosis, neoangiogenesis and peritoneal dysfunction [1]. PD represents an essential risk factor for nosocomial infections along with diabetes, hemodialysis, medical implants, endotracheal intubation, indwelling catheters, immunosuppressive therapy and trauma [2]. *Staphylococcus aureus* (*S. aureus*) infections occur frequently in these at-risk patient groups, making it necessary to develop effective preventive measures. Mouse models for infectious diseases caused by *S. aureus* provide experimental proof for the molecular basis of pathogenesis and the molecular mechanisms of actions of the potential anti-infective agents [2]. Intraperitoneal staphylococcal contamination is used in studies investigating the efficacy of potential drugs and vaccines due to the convenience of administering large inoculations and obtaining highly reproducible data [3]. Recognizing the global spread of antibiotic resistance, methicillin-resistant *S. aureus* (MRSA) remains one of the most lethal antibiotic-resistant pathogens. At the same time, some methicillin-sensitive *S. aureus* (MSSA) strains are also of substantial clinical importance because of their high virulence and cause of fatal infections [4]. Novel approaches to controlling *S. aureus* include targeting a known virulence determinant influencing pathogenicity or aiming to simultaneously eliminate several virulence factors [4]. Targeting adhesins, toxins, secretion systems, siderophores, immune evasion and modulation factors, and biofilm formation factors advances the basis for antivirulent therapies that may complement available antibiotic treatment or could be used as prophylaxis in high-risk patients [5].

Recently we demonstrated attenuation of *S. aureus* bacterial adhesion to eukaryotic cells induced by the pure pentapeptide Leu-Pro-Arg-Asp-Ala (LPRDA) and caused by defective cell wall-anchored (CWA) protein exposition [6]. We also revealed that both prokaryotic and eukaryotic cell lines remained viable under LPRDA influence as well as showing LPRDA *S. aureus* biofilm inhibitory properties [6]. In the present work, a comparative assessment of the bacterial load in the internal organs of experimental animals was performed for the first time in a mouse model of peritonitis when they were challenged with a sublethal dose of *S. aureus* during prophylactic and therapeutic administration of the individual pentapeptide LPRDA. The effect of the single pentapeptide LPRDA on the expression of the *S. aureus* virulence factors alpha-hemolysin (Hla) and the in vitro effect of LPRDA on the minimum inhibitory concentrations of clinically relevant antibiotics were also investigated.

## 2. Results

### 2.1. LPRDA Synthesis and Characterization

The pentapeptide LPRDA, consisting of five linked L-amino acid residues (Leu-Pro-Arg-Asp-Ala), is a partial structural analog of the LPXTG sorting signal. It was synthesized via solid-phase peptide synthesis using the 9-fluorenylmethoxycarbonyl chemistry method with a purity of 99+% [6]. The secondary structure of LPRDA was studied using nuclear magnetic resonance (NMR) spectroscopy [7], confirming the homogeneity and assigned formula of the pentapeptide sample used for the biological experiments. The physicochemical properties of LPRDA are presented in Table 1. Corresponding values were obtained from the appropriate web services [8,9] and our previous work [10].

### 2.2. LPRDA Lethal Dose (LD) Determination

The in vivo experiments that were performed demonstrated that all the laboratory animals given different doses of LPRDA (n = 3 for each dose of 250, 500 and 1000 mg/kg), administered intravenously (i.v.), survived for at least 7 days. Accordingly, the LD_50_ of LPRDA exceeded 1000 mg/kg.

### 2.3. Induction of Acute Murine Peritonitis

**Figure 1:**
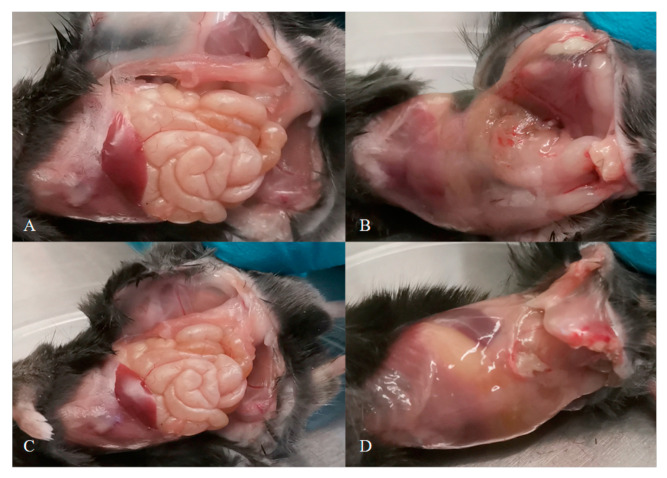
Abdominal cavities of *S. aureus*-infected mice on the 7th day with pre-administration of LPRDA (**A**,**C**) and the non-treated control group (**B**,**D**).

The survival rates of mice in both groups (n = 5 in each group) with and without intraperitoneal administration of LPRDA were 100% for 7 days when peritonitis was induced with a reference strain of *S. aureus* ATCC 29213 (total dose 4 × 10^7^ CFU). In animals pre-injected with 250 mg/kg LPRDA, the intestinal loops and liver lobe were clearly visible in the abdominal cavity, and the peritoneal sheets were not fused with the internal organs (Figure 1A,C). Mice from the control group injected with NaCl physiological solution had characteristic macroscopic signs of acute nonspecific peritonitis: thickening and turbidity of visceral and parietal peritoneal sheets with petechial hemorrhages; and turbidity and stickiness of serous membranes. The abdominal cavity organs in the control group were poorly visualized during examination due to adhesion of the visceral peritoneal sheet (Figure 1B,D).

### 2.4. Preventive Administration of LPRDA in the Model of Induced Murine Peritonitis

**Figure 2:**
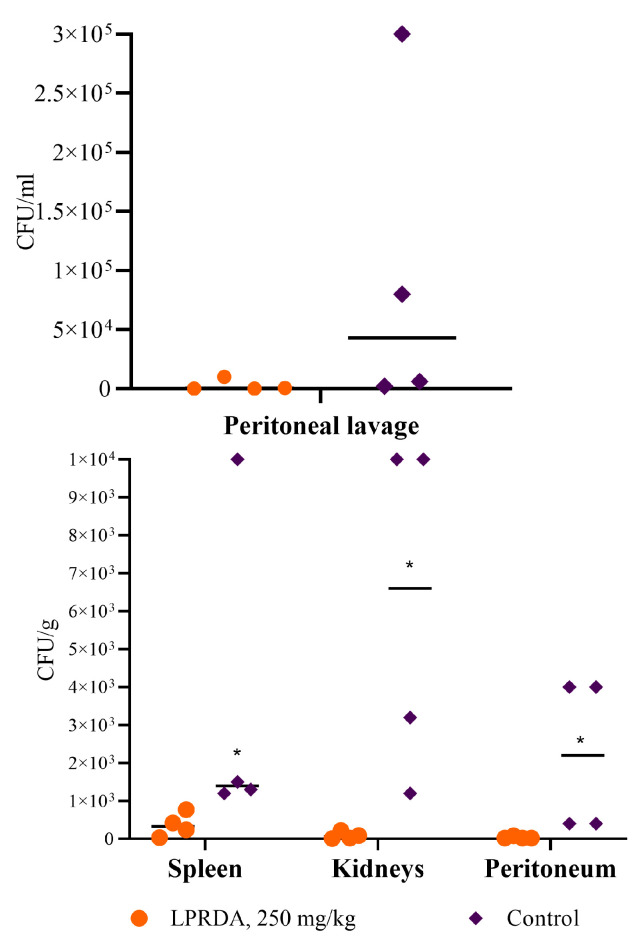
Bacterial burden of the internal organs and peritoneal lavage of mice with modeled *S. aureus* peritonitis. * *p* < 0.05.

The bacterial burden of the internal organs in animals receiving a single intraperitoneal injection of 250 mg/kg LPRDA 5 min prior to *S. aureus* infection was significantly reduced compared to the control group (*p* = 0.03, Figure 2). The single injection of LPRDA decreased the median bacterial contamination of peritoneal lavage from 43,000 (3000; 245,000) to 250 (0; 7625). The highest level of contamination in the experimental group was found in the spleen—330 (85; 682)—while in the control group it was found in the kidney—6600 (1700; 10,000) (Figure 2). However, the intra-group differences in bacterial contamination of the analyzed organs were not statistically significant.

### 2.5. Therapeutic Administration of LPRDA in the Model of Induced Murine Peritonitis

**Figure 3:**
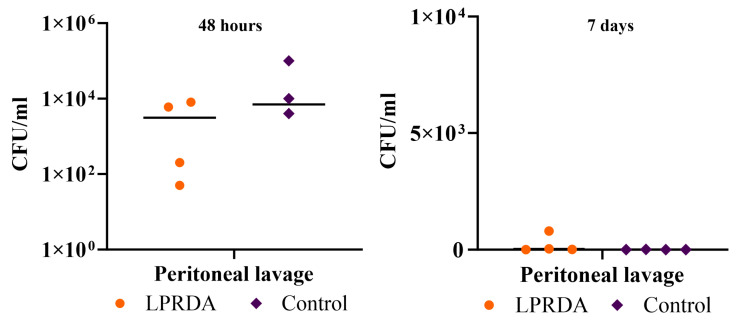
Bacterial load of peritoneal lavage from laboratory animals 48 h and 7 days after initiation of bacterial peritonitis caused by MSSA 29213.

**Figure 4:**
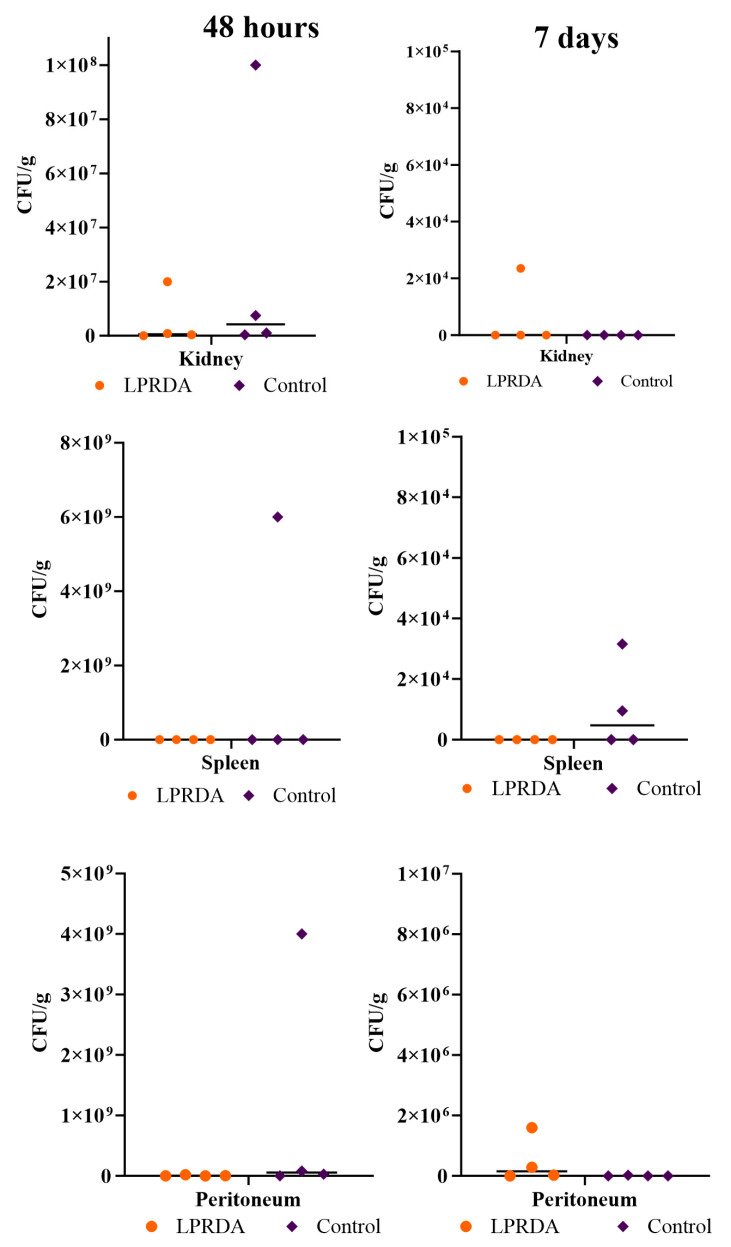
Bacterial load of experimental animal organs 48 h and 7 days after initiation of bacterial peritonitis caused by MSSA 29213.

The survival rates of animals in the model of induced murine peritonitis [3] caused by the *S. aureus* ATCC 29213 (MSSA) strain with the prolonged administration of the LPRDA pentapeptide after 48 h and at day 7 of infection with 2.4 × 10^8^ CFU were 100% in both the experimental and control groups. Comparative analysis of the bacterial contamination of the internal organs and peritoneal lavage (Figure 3 and Figure 4) after 48 h revealed no statistically significant differences between the experimental and control groups. However, the bacterial contamination of the peritoneum was lower in the animals receiving LPRDA compared to the control group: 1.9 × 10^6^ (6 × 10^5^; 1 × 10^7^) CFU/g and 2 × 10^7^ (8.7 × 10^6^; 4.5 × 10^9^) CFU/g (*p* = 0.34), respectively.

On day 7, the bacterial counts of the biomaterials studied were generally lower than on day 2 of infection. Differences in the median bacterial counts between the LPRDA experimental and control groups were not statistically significant: 25 (2.5; 610) and 0 (0; 7.5) CFU/mL (*p* = 0.99), and 1.5 × 10^5^ (6 × 10^3^; 1.2 × 10^6^) and 0 (0; 2 × 10^4^) CFU/g (*p* = 0.08) for peritoneal lavage, respectively. The MSSA strain was isolated from the peritoneum in three out of four cases in the experimental group and in one out of four cases in the control group, and from the peritoneal lavage in three and one cases, respectively. No MSSA culture was isolated from the spleen of any of the animals in the experimental group, while in the control group, negative cultures were obtained in only two cases, the other two animals having spleen counts of 9 × 10^3^ and 3 × 10^4^ CFU/g. All the animals in the control group and three individuals in the experimental group also had no growth of MSSA from the kidney. In one case in the experimental group, the bacterial load was 2 × 10^4^ CFU/g.

**Figure 5:**
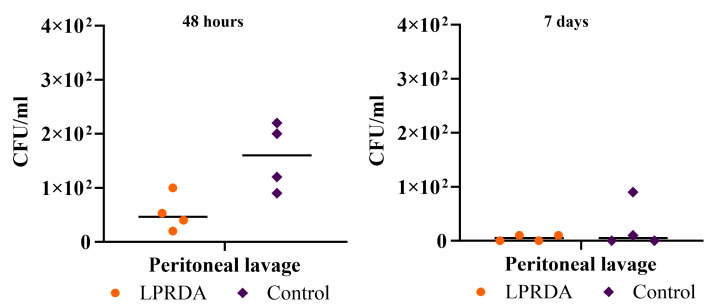
Bacterial contamination of peritoneal lavage in 48 h and 7 days after the inducing of bacterial peritonitis caused by MRSA 43300.

**Figure 6:**
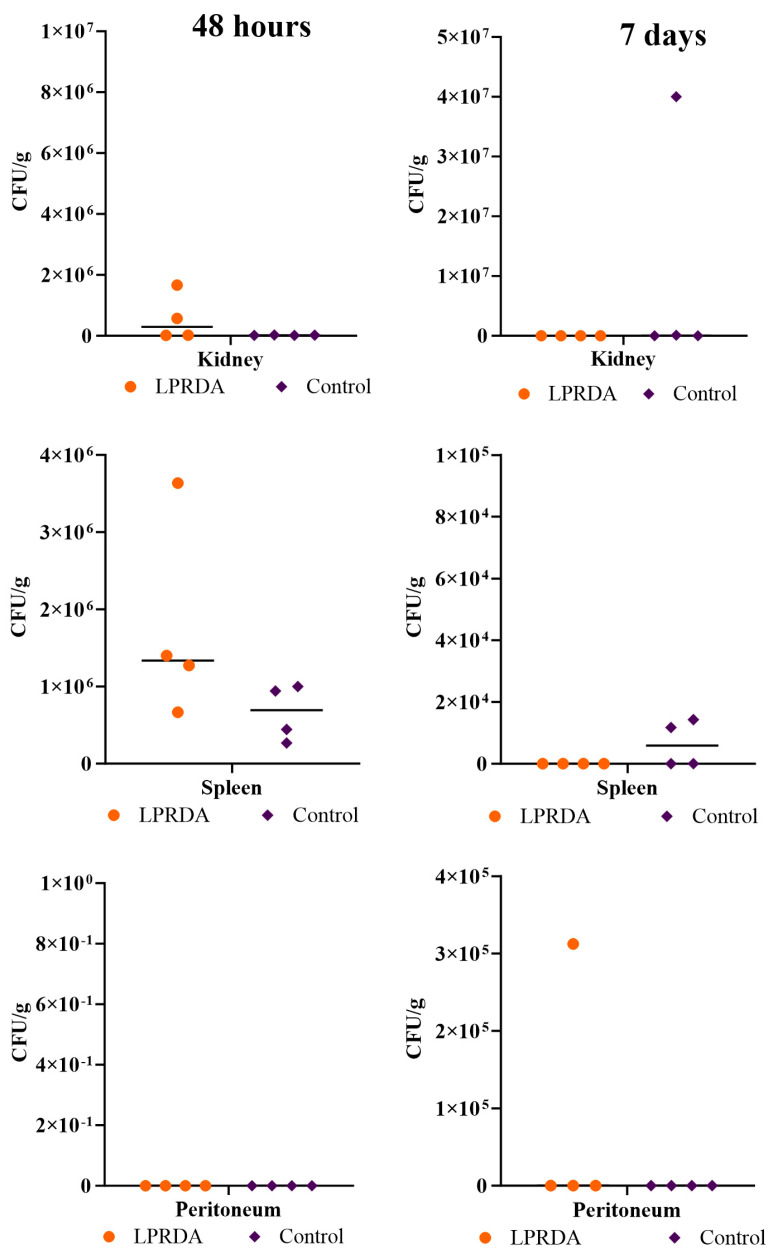
Bacterial contamination of the organs at 48 h and 7 days after the inducing of bacterial peritonitis caused by MRSA 43300.

The survival rates of animals in the model of bacterial peritonitis caused by 2.4 × 10^8^ CFU of *S. aureus* ATCC 43300 (MRSA) at 48 h and on day 7 after infection were 100% in both groups. There were no statistically significant differences in the indicated terms in the contamination of internal organs and peritoneal lavage of laboratory animals (Figure 5 and Figure 6). It is noteworthy that in contrast to the experiment with MSSA, no infectious MRSA strain was isolated from the peritoneum of any animal after 48 h. Peritoneal lavage infectivity (46.5 (25; 89) and 1.6 × 10^2^ (98; 2.1 × 10^2^) CFU/mL for the MRSA experimental and control groups, respectively) was lower than in the corresponding MSSA experiment (3.1 × 10^3^ (88; 7.5 × 10^3^) and 7 × 10^3^ (1 × 10^3^; 7.8 × 10^4^) CFU/mL (*p* = 0.9), respectively).

On the 7th day of modeling peritonitis caused by MRSA, the infectious agent was isolated from the peritoneum at the level of 3.2 × 10^5^ CFU/g in only one animal after the prolonged LPRDA administration; the rest of the samples of the peritoneum and other organs did not show bacterial growth. In the control group, no growth was obtained from the peritoneum, but MRSA growth was obtained from the spleen and kidney in two cases. Single colonies of MRSA were isolated from the peritoneal lavage of two animals in the experimental group and one individual in the control group. Another animal in the control group had moderate growth of the pathogen from the lavage (Figure 6).

### 2.6. LPRDA Inhibition of S. aureus α-Hemolysin Expression

**Figure 7:**
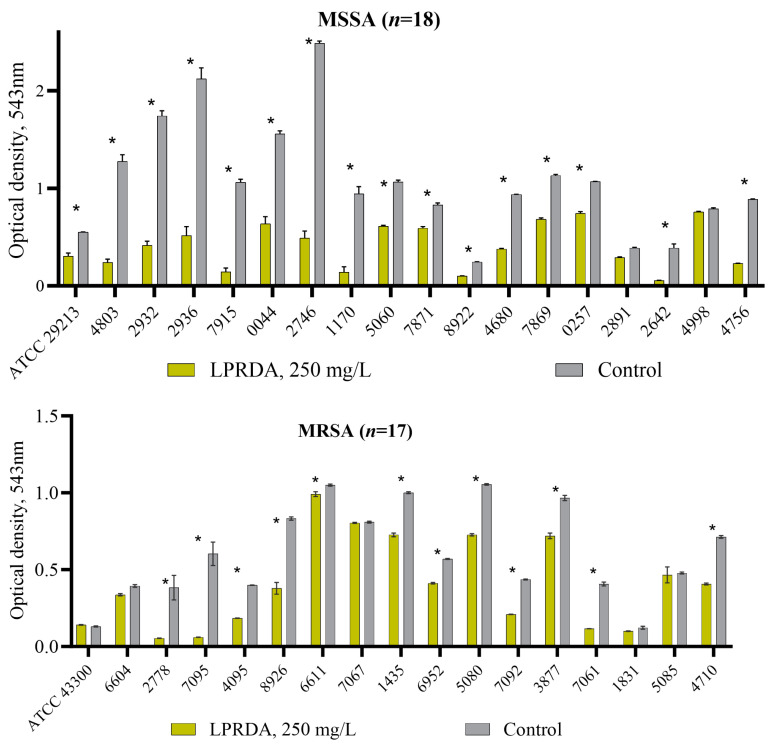
Level of *S. aureus* hemolysis of rabbit erythrocytes under LPRDA bacterial treatment. * *p* < 0.05.

Thirty-five strains of *S. aureus* (two reference and thirty-three clinical strains) were tested for inhibition of rabbit erythrocytes hemolysis [11] under the preliminary treatment of *S. aureus* strains with LPRDA. All the studied *S. aureus* strains were characterized by α-hemolysin (Hla) production. The inhibitory effect of LPRDA was registered in 80% of the studied *S. aureus* strains (Figure 7). Thus, out of 18 MSSA isolates, 16 species had a statistically significant decrease in Hla production, including the reference MSSA 29213 (Table 2). MRSA strains were less affected by LPRDA and out of the studied 17 cultures a decrease in *S. aureus* hemolytic activity was registered in 12 species (Table 2).

### 2.7. Interaction of LPRDA with Antibiotics

The co-incubation of *S. aureus* with LPRDA and cefazolin did not change the minimal inhibitory concentration (MIC) of the antibiotic against MSSA ATCC 29213. Bacterial growth was recorded in wells with cefazolin concentrations below the MIC (0.5 mg/L) regardless of the LPRDA concentration. Against MRSA ATCC 43300 in the presence of LPRDA, a 2-fold increase in the MIC of vancomycin from 0.5 mg/L to 1 mg/L was detected for all the tested LPRDA concentrations.

## 3. Discussion

Peritoneal *S. aureus* infection is common in patients on continuous ambulatory peritoneal dialysis (CAPD) and manifests as inflammation of the peritoneal catheter exit site and tunnel, peritonitis and peritoneal abscesses. High doses of cefazolin or vancomycin for several weeks are used to treat these conditions, but resolution is observed in only 45–73% of cases, posing a significant risk to patients undergoing PD [12].

The increasing prevalence of multidrug-resistant strains necessitates the development of unconventional therapeutics to control bacteria and overcome resistance. Cell wall-anchored (CWA) proteins and toxins are among the most important and recognized *S. aureus* virulence targets, as determined by their distribution and contribution to disease [5]. The virulence and disease-promoting properties of *S. aureus* CWA proteins have been demonstrated by inactivating the corresponding bacterial gene expression and subsequent studying the infectious processes of the defective pathogens [13]. A number of *S. aureus* CWA proteins are anchored to the bacterial cell wall by the sortase A (SrtA) enzyme [14]. The influence of sortases and their inhibition of the in vivo pathogenesis of pneumonia, septic endocarditis, septic arthritis, mastitis and cutaneous and gastrointestinal infections has previously been demonstrated, with an apparent reduction in incidence rates achieved by suppressing sortase functionality [15]. Many SrtA inhibitors have been synthesized [16,17,18,19], but in vivo efficacy evaluation and follow-up of in-depth studies are required for the introduction of treatment options based on these agents to clinical practice. The pentapeptide Leu-Pro-Arg-Asp-Ala (LPRDA) is one of the most studied *S. aureus* SrtA competitive inhibitors [19]. Although the SrtA inhibitory activity of the pentapeptide has been reported differently by investigators [20,21], LPRDA induced a reduction in staphylococcal adhesion to eucaryotic cells [6] and demonstrated a therapeutic effect against *S. aureus*-induced mastitis in a mouse model [20]. The current study has assessed the protective and therapeutic effects of LPRDA in a mouse model of acute peritoneal *S. aureus* infection.

In contrast to Wang et al., who used LPRDA modified by PEG2000 and amide modifications at the N- and C-termini, respectively [20], we utilized an individual sample of the LPRDA pentapeptide of high purity (>99%). Therefore, the C57BL/6 mice were given a 250 mg/kg dose of LPRDA (experimental group) or placebo (control group), and subsequently they were treated with a sublethal dose of *S. aureus* ATCC 29213 (MSSA) strain (4 × 10^7^ CFU) by intraperitoneal injection. Macroscopic patterns on day 7 of infection demonstrated the prevention of *S. aureus* colonization in the abdominal cavities of the mice with preliminary administration of LPRDA (Figure 1). To examine if LPRDA enhances bacterial clearance in the murine peritonitis model, C57BL/6 mice were administered 250 mg/kg dose of LPRDA (experimental group) or placebo (control group), and subsequently they were treated by intraperitoneal injection with 4 × 10^7^ CFU of *S. aureus* ATCC 29213 (MSSA). On day 2 following the intraperitoneal challenge, the infected mice were euthanized, and the internal organs were homogenized. The median staphylococcal load in the tissues and organs of the mice pretreated with LPRDA displayed a 10–100-fold reduction in peritoneal lavage, peritonium and spleen species and a 1000-fold reduction in kidney species compared to the vehicle mice group (Figure 2). The results for bacterial burden reduction are consistent with our previous observations of the reduced adhesion of LPRDA-pretreated *S. aureus* to Vero cells [6].

To evaluate the therapeutic options of the LPRDA administration, the animals were infected with MSSA and MRSA ATCC strains, with a sublethal dose of 2.4 × 10^8^ CFU in each case. LPRDA (250 mg/kg) was administered intravenously to animals in the experimental group at 24 h intervals post-infection. On days 2 and 7, selected animals were euthanized, and the internal organs were harvested, homogenized and subjected to CFU counting. Accordingly, therapeutic administration of the individual LPRDA did not confer protective efficacy in the peritoneal challenge model. On the other hand, a prolonged intravenous administration of the pentapeptide has confirmed its tolerability and safety in vivo, which was also demonstrated in the LD determination study (Section 2.2).

Multiple virulence factors appear to be required for the pathogenesis of different *S. aureus* infectious diseases, and these may differ between disease types. Extracellular toxins are deeply involved in the pathogenesis of *S. aureus* infections. The inhibition of toxins is thought to exert less selective pressure for the development of resistance than killing or preventing bacterial growth. Alpha-hemolysin (Hla), expressed by most clinical isolates of *S. aureus*, is a major extracellular toxin that contributes to pneumonia, dermonecrosis, endocarditis, sepsis and peritonitis [3,22]. Hla forms a heptameric pore to penetrate the cell membrane, causing cell injury and death. We decided to evaluate the production of Hla by *S. aureus* strains after their incubation with the studied LPRDA and rather unexpectedly discovered a significant decrease in rabbit erythrocyte hemolysis for those treated with LPRDA ATCC and clinical bacterial samples. A spectrophotometric assay was used to quantify the release of hemoglobin from erythrocytes in response to hemolysin (Figure 7). The results show that 80% of the studied *S. aureus* strains, both MSSA and MRSA, are susceptible to LPRDA and have reduced Hla expression (Table 2). To the best of our knowledge, the LPRDA pentapeptide is the first representative of the class of oligopeptides with dual antisortase and antitoxin activities. An analogous dual inhibitory effect has been reported for chalcone [23], isosakuranetin [24] and, seemingly, for chlorogenic acid [25], but subsequent development of these small-molecule agents is questionable due to absorption, distribution, metabolism, elimination and toxicity (ADMET) issues and potential bactericidal activity.

Antibacterial in vivo studies of sortase A inhibitors have been performed in a systemic infection model using intravenous staphylococcal challenge [25,26,27,28,29] and in a pneumonia model using inhalation challenge [30,31,32,33,34,35,36]. Although some promising results have been obtained with synthetic and natural SrtA inhibitors for the treatment of *S. aureus* model infections, in several cases the SrtA inhibitors have worked as adjuvants to clinically used antibiotics [30,31,32,34]. As we did not receive strong evidence for the therapeutic activity of LPRDA in the induced *S. aureus* peritonitis (Section 2.5), and to plan future combined administration, we studied antibacterial drug interactions with the LPRDA pentapeptide (Section 2.7). The MIC of cefazolin against MSSA ATCC 29213 was not changed in the presence of LPRDA, while the similar parameter of vancomycin against MRSA ATCC 43300 doubled from 0.5 mg/L to 1 mg/L. It appears that the desired curative effect can be achieved through the combined use of the studied agents.

## 4. Materials and Methods

### 4.1. Reagents, Cell Cultures and Strains

The pentapeptide LPRDA was synthesized using the solid-phase peptide 9-fluorenylmethoxycarbonyl chemistry method [6]. This produces a colorless light solid, soluble in water, with 99.15% de (HPLC, Kinetex EVO C18 (Phenomenex, Castel Maggiore, Italy), *t_R_* 8.18 min). The HRMS (ESI, *m*/*z*): [M + H]^+^ was calculated for C_24_H_42_N_8_O_8_, 571.3204; we found 571.3188.

The *Staphylococcus aureus* strains used in the study were methicillin-susceptible *Staphylococcus aureus* (MSSA) ATCC 29213, methicillin-resistant *Staphylococcus aureus* (MRSA) ATCC 43300 and several MSSA and MRSA clinical strains. Identification of the strains used for the infection and isolated from animal tissues was performed by MALDI-TOF-MS using the FlexControl system and MBT Compass 4.1 software (Bruker Daltonics GmbH & Co. KG, Bremen, Germany).

### 4.2. Animal Care and Compliance Statement

The animal experiments were conducted with 4–6-week-old C57BL/6 mice according to the guidelines of the Declaration of Helsinki (EU Council Directive 86/609/EEC) after the approval by the Local Ethics Committee of the Vreden National Medical Research Center of Traumatology and Orthopedics (St. Petersburg, Russia). The mice were maintained in cages with Lignocel (JRS, Rosenberg, Germany) bedding. They received filtered water and a standard chow diet PK-120 (Melkombinat, Russia).

### 4.3. LPRDA Lethal Dose (LD) Determination

LD_50_ of LPRDA was determined using the up-and-down method according to the guidelines by the Organization for Economic Co-operation and Development (OECD) [37]. The sample of LPRDA was dissolved in 0.9% NaCl solution. For sedation, the animals were intraperitoneally (i.p.) injected with 80 μL of ketamine (50 mg/mL) and sibasone (5 mg/mL) mixed in the ratio of 1:1. Then, mice (n = 3) were injected with LPRDA at the initial dose of 250 mg/kg once intravenously (i.v.) into the retro-orbital plexus with a syringe and a 27 G needle. After 48 h, the procedure was repeated with a new group of animals (n = 3), increasing the dose of LPRDA 2-fold. The animals were monitored for 7 days. In total there were three series of experiments with LPRDA doses of 250, 500 and 1000 mg/kg, with three animals in each.

### 4.4. Induction of Murine Peritonitis

All the animal experiments in our study were performed in a model of induced murine peritonitis [3]. The reference strain of *S. aureus* ATCC 29213 was cultivated in a shaker (Cryste Novapro, Seoul, Republic of Korea) at 37 °C and at 100 rpm for 20 h. Then, 1 mL of bacterial suspension was added to 10 mL of fresh sterile culture medium and incubated until reaching the optical density (OD_540_) of 0.5 on the McFarland scale. The bacterial suspension was centrifuged for 10 min at 3000× *g* (Eppendorf, Germany) and washed three times with a sterile solution of 0.9% NaCl. The sediment was resuspended to a concentration of 8 × 10^8^ CFU/mL. Bacterial counts were confirmed by counting colony-forming units on Mueller–Hinton agar plates.

For the induction of peritonitis, the mice were injected i.p. with 250 mg/kg of LPRDA into the left lower quadrant of the abdomen (n = 5, experimental group) or 100 μL of 0.9% NaCl (n = 5, control group). After 5 min, the animals were injected on the same side with 50 μL of 8 × 10^8^ CFU/mL of *S. aureus* ATCC 29213 overnight culture (total dose 4 × 10^7^ CFU). The survival of the animals was observed for 7 days. Then they were sacrificed by overdose (1.0 mL) of 10% sodium thiopental. The macroscopic state of the internal organs in the selected animals was observed.

### 4.5. Preventive Administration of LPRDA in the Model of Induced Murine Peritonitis

Determination of the bacterial burden of the internal organs was carried out after the initial preventive i.p. injection of LPRDA. Initially, 250 mg/kg of LPRDA peptide was injected into the left lower quadrant of the mouse abdomen in the experimental group (n = 4) using a syringe with a 27G needle. Animals in the control group (n = 4) received 100 μL of 0.9% NaCl. After 5 min, for the induction of peritonitis, 50 μL of 8 × 10^8^ CFU/mL *S. aureus* ATCC 29213 overnight culture (total dose 4 × 10^7^ CFU) was injected i.p. into the same side of the abdomen. After 48 h, the animals were taken out of the experiment. Then, under sterile conditions, the skin was removed from the abdomen and peritoneal lavage with 3 mL 0.9% NaCl was performed. The injected NaCl solution was evenly distributed in the peritoneal cavity and aspirated through a 27G needle. With sterile tweezers, a part of the peritoneum, the spleen and left kidney were harvested for quantitative bacteriological study.

The obtained organ samples were weighed and homogenized in 1 mL of 0.9% NaCl. The homogenisates and peritoneal lavage were titrated to a dilution of 10^7^ and 100 µL of the obtained suspensions of each dilution were sown on the surface of Mueller–Hinton agar by the lawn method. The dishes were incubated for 24 h at 37 °C, after which colony counts were performed and the contamination per 1 g of organ or 1 mL of peritoneal lavage was calculated. Identification of the grown colonies was performed by MALDI-TOF-MS and matched the original *S. aureus* ATCC 29213.

### 4.6. Therapeutic Administration of LPRDA in the Model of Induced Murine Peritonitis

The effect of the prolonged 2- and 7-day i.v. administration of LPRDA on the internal organ bacterial burden was studied. Firstly, all the mice (n = 16) were injected i.p. with 30 µL of 8 × 10^9^ CFU/mL suspension of MSSA ATCC 29213 (total dose 2.4 × 10^8^ CFU). Then, 250 mg/kg of LPRDA pentapeptide was administered into the lateral tail vein every 24 h in the animals in the experimental group (n = 8) and 50 μL of 0.9% NaCl into the animals in the control group (n = 8). After 48 h and on day 7, half of the animals (n = 8) at each time point were sacrificed for analysis. The procedures for peritoneal lavage, organ harvesting and quantitative bacteriological study were performed in the same way as described in Section 4.5. Identification of the grown colonies was performed by MALDI-TOF-MS and matched the original *S. aureus* ATCC 29213.

In the next experiment, all the animals (n = 16) were injected i.p. with 30 µL of 8 × 10^9^ CFU/mL suspension (total dose 2.4 × 10^8^ CFU) of the overnight MRSA ATCC 43300 culture. Then, 250 mg/kg of LPRDA pentapeptide were administered into the lateral tail vein every 24 h. After 48 h and on day 7, mice (n = 8) at each time point were also sacrificed. The procedures for peritoneal lavage, organ harvesting and quantitative bacteriological study were performed the same way as described in Section 4.5. Identification of the grown colonies was performed by MALDI-TOF-MS and matched the original *S. aureus* ATCC 43300.

### 4.7. Hemolysis Detection Assay

The inhibitory effect of LPRDA on α-toxin production by *S. aureus* was evaluated according to the method of Tao et al. with minimal modification [11]. First, 200 µL of *S. aureus* bacterial suspension was added to 1 mL of Mueller–Hinton broth (MHB) containing 250 mg/L LPRDA in the experimental group, and only MHB in the control group. The tubes were incubated for 24 h, then centrifuged for 5 min at 5000× *g* rpm. Next, 100 μL of supernatant was mixed with 875 μL of DPBS and 25 μL of rabbit erythrocytes and incubated at 37 °C for 30 min. Triton X-100 was used as a positive control and DPBS was used as a negative control. The mixture was then centrifuged for 3 min at 4000× *g* rpm and 4 °C. The supernatant obtained was transferred to the wells of a 96-well plate and the optical density (OD) at 543 nm was measured on a spectrophotometer.

### 4.8. LPRDA Influence on Antibiotic Properties of Antibacterial Drugs

The checkerboard method [38] was used to evaluate antibacterial drug (AD) interaction with LPRDA pentapeptide. The minimum inhibitory concentration (MIC) of cefazolin against a reference strain of MSSA and vancomycin against a reference strain of MRSA was determined using serial dilutions. Next, 100 µL of MHB containing a specific concentration of antibiotic cefazolin (0.052 to 8 mg/L), vancomycin (0.06 to 2 mg/L) and 100 µL of MHB with LPRDA (50–250 mg/L) were added to the wells of a flat-bottomed 96-well plate. Then, 50 μL of bacterial suspension with an OD of 0.5 on the McFarland scale was added. For the positive control, 200 μL of MHB and 50 μL of bacterial suspension were used. For the control, we used 100 µL of MHB with antibiotic, 100 µL of MHB and 50 µL of bacterial suspension. The plate was incubated for 24 h at 37 °C and then the results were recorded.

### 4.9. Statistical Analysis

Statistical analysis was performed using GraphPad Prism 9.0 (GraphPad Software, Boston, MA, USA). The results of the bacterial burden count are presented as medians with their lower and upper quartiles. Differences between the groups were assessed using the Mann–Whitney test. The results of the hemolytic assay are presented as mean with the standard deviation. Differences between the groups were assessed using one-way ANOVA analysis of variance. All the tests were considered statistically significant at *p* < 0.05.

## 5. Conclusions

To summarize, the pentapeptide Leu-Pro-Arg-Asp-Ala (LPRDA) reduced the *S. aureus* virulence in vivo and provided significant protection against *S. aureus* peritoneal infection in mice. The incubation of LPRDA with reference and clinical MSSA and MRSA strains decreased the hemolytic activity of *S. aureus*. The multitargeted pathogen interactions, in vivo tolerability and safety of LPRDA allow its further evaluation as a potential antibacterial agent with a novel mechanism of action.

## Figures and Tables

**Table 1:** Physicochemical properties of the LPRDA pentapeptide.

Net Charge at pH 7.4 [8]	GRAVY * [8]	Isoelectric Point [8]	Dipole Moment (Debyes) [9]	TPSA ** (Å^2^) [10]	Molecular Weight (g/mol)
0	−0.80	5.84	74	279.15	570.64

* Grand Average of Hydropathy (GRAVY). ** Topological Polar Surface Area (TPSA).

**Table 2:** Natural and experimental (after LPRDA treatment) *S. aureus* hemolytic activities (OD_543_).

*S. aureus* Strain	Experimental *	Control *	*p*
MSSA ATCC 29213 **	0.304 ± 0.032	0.551 ± 0.003	<0.0001
**MSSA 4803**	0.239 ± 0.035	1.277 ± 0.067	<0.0001
**MSSA 2932**	0.418 ± 0.040	1.744 ± 0.051	<0.0001
**MSSA 2936**	0.515 ± 0.093	2.125 ± 0.110	<0.0001
**MSSA 7915**	0.143 ± 0.040	1.064 ± 0.029	<0.0001
**MSSA 0044**	0.635 ± 0.075	1.559 ± 0.031	<0.0001
**MSSA 2746**	0.491 ± 0.072	2.486 ± 0.024	<0.0001
**MSSA 1170**	0.143 ± 0.054	0.944 ± 0.073	<0.0001
**MSSA 5060**	0.612 ± 0.008	1.066 ± 0.019	<0.0001
**MSSA 7871**	0.592 ± 0.017	0.829 ± 0.020	<0.0001
**MSSA 8922**	0.101 ± 0.002	0.245 ± 0.003	0.0007
**MSSA 4680**	0.378 ± 0.006	0.939 ± 0.001	<0.0001
**MSSA 7869**	0.683 ± 0.014	1.132 ± 0.010	<0.0001
**MSSA 0257**	0.744 ± 0.017	1.070 ± 0.002	<0.0001
MSSA 2891	0.291 ± 0.006	0.389 ± 0.005	0.0675
**MSSA 2642**	0.055 ± 0.002	0.390 ± 0.040	<0.0001
MSSA 4998	0.759 ± 0.004	0.790 ± 0.009	0.9995
**MSSA 4756**	0.230 ± 0.003	0.888 ± 0.004	<0.0001
MRSA ATCC 43300	0.141 ± 0.003	0.131 ± 0.003	>0.9999
MRSA 6604	0.337 ± 0.008	0.394 ± 0.009	0.0631
**MRSA 2778**	0.054 ± 0.002	0.383 ± 0.080	<0.0001
**MRSA 7095**	0.060 ± 0.001	0.603 ± 0.076	<0.0001
**MRSA 4095**	0.184 ± 0.001	0.399 ± 0.002	<0.0001
**MRSA 8926**	0.379 ± 0.038	0.832 ± 0.010	<0.0001
**MRSA 6611**	0.991 ± 0.016	1.050 ± 0.007	0.0444
MRSA 7067	0.805 ± 0.004	0.809 ± 0.006	>0.9999
**MRSA 1435**	0.726 ± 0.012	1.001 ± 0.006	<0.0001
**MRSA 6952**	0.412 ± 0.005	0.570 ± 0.003	<0.0001
**MRSA 5080**	0.727 ± 0.008	1.054 ± 0.005	<0.0001
**MRSA 7092**	0.210 ± 0.002	0.436 ± 0.003	<0.0001
**MRSA 3877**	0.720 ± 0.018	0.966 ± 0.017	<0.0001
**MRSA 7061**	0.116 ± 0.001	0.407 ± 0.012	<0.0001
MRSA 1831	0.100 ± 0.002	0.122 ± 0.009	0.991
MRSA 5085	0.466 ± 0.052	0.478 ± 0.006	>0.9999
**MRSA 4710**	0.407 ± 0.006	0.713 ± 0.009	<0.0001

* OD_543_ (mean ± SD). ** *S. aureus* strains with statistically significant hemolysis decreasing are indicated with bold.

## Data Availability

The generated research data are available from the authors on request.
